# The Use of TB-Specific Antigen/Phytohemagglutinin Ratio for Diagnosis and Treatment Monitoring of Extrapulmonary Tuberculosis

**DOI:** 10.3389/fimmu.2018.01047

**Published:** 2018-05-14

**Authors:** Feng Wang, Jing Yu, Yu Zhou, Ying Luo, Shiji Wu, Min Huang, Botao Yin, Jing Huang, Liyan Mao, Ziyong Sun

**Affiliations:** Department of Laboratory Medicine, Tongji Hospital, Tongji Medical College, Huazhong University of Science and Technology, Wuhan, China

**Keywords:** extrapulmonary tuberculosis, T-SPOT.TB, tuberculosis-specific antigen/phytohemagglutinin ratio, diagnosis, immunosuppression

## Abstract

Extrapulmonary tuberculosis (EPTB) has become more common in recent years; however, the diagnosis of EPTB remains a challenge. In this study, we analyzed the performance of the ratio of TB-specific antigen (TBAg) to phytohemagglutinin (PHA) (TBAg/PHA ratio) in T-SPOT.TB (T-SPOT) assay for diagnosis and treatment monitoring of EPTB. Between 2012 and 2017, 734 EPTB patients were diagnosed and recruited from Tongji hospital, and 1,137 suspected EPTB patients who had other diagnoses were recruited as non-EPTB controls. To validate the study, another small group of EPTB patients and non-EPTB controls were recruited from Sino-French New City Branch of Tongji Hospital. The positive rate of peripheral blood T-SPOT in EPTB and non-EPTB were 88.15 and 32.28%. In T-SPOT positive patients, the direct T-SPOT results had limited value in distinguishing these two conditions. A further calculation of the TBAg/PHA ratio of T-SPOT showed improved performance in each form of EPTB. If using 0.20 as the threshold value of the TBAg/PHA ratio, the pooled sensitivity and specificity were 70.79 and 91.55% in distinguishing EPTB from non-EPTB. The validation results showed a better performance of the TBAg/PHA ratio in distinguishing these two conditions, with a sensitivity and specificity of 81.82 and 97.56%, respectively. Comparing with directly using T-SPOT results, the TBAg/PHA ratio was less affected by immunosuppression. Furthermore, PHA value reflected immunosuppression and could help to judge the credibility of T-SPOT results in EPTB patients with different immune status. The TBAg/PHA ratio was significantly decreased during anti-tuberculosis (TB) treatment, which suggests that it can also be used to monitor therapeutic efficacy. These data provide new insights into the role of T-SPOT assay in TB disease, and the TBAg/PHA ratio might be a useful tool for diagnosis and treatment monitoring of EPTB.

## Key Points

- The TBAg/PHA ratio of T-SPOT.TB assay is less affected by immunosuppression.
- Calculation of the TBAg/PHA ratio has the potential in the diagnosis and treatment monitoring of EPTB.

## Introduction

Extrapulmonary tuberculosis (EPTB) has become more common in recent years. It ranges from 10 to 50% of all cases of tuberculosis (TB) and still increases in some countries (1, 2). Patients with EPTB do not receive specific attention in international TB control strategies (3). However, EPTB contributes significantly to TB-related morbidity and can cause complications, lifelong sequelae, and disabilities (4). Due to the heterogeneity in clinical manifestations, the pauci-bacillary nature of disease, and the need for invasive procedures to obtain appropriate samples, EPTB remains an important diagnostic and therapeutic challenge (4, 5).

Currently, there is no one available test that appears to be suitable for the diagnosis of EPTB. The sensitivity of Acid-fast Bacillus (AFB) smear is low among patients with pulmonary TB (6), and it is further decreased in the diagnosis of EPTB because of the pauci-bacillary nature of disease (5). *Mycobacterium tuberculosis* (Mtb) culture faces the same dilemma as AFB smear and is also limited by a long turnaround time (7). The polymerase chain reaction-based Xpert MTB/RIF assay also has poor sensitivity under low bacterial loads and cannot distinguish live and nonviable Mtb contributions (8, 9). A recent WHO policy update acknowledged the low quality of evidence supporting the use of Xpert MTB/RIF to diagnose EPTB (10, 11). Very recently, a new non-invasive method which uses NanoDisk-MS to quantification of circulating Mtb antigen peptides shows high sensitivity and specificity in the diagnosis of both pulmonary TB and EPTB (12). However, the performance of this method in the diagnosis of EPTB should be validated due to a very small number of cases.

Interferon-gamma release assays (IGRAs) which measure *ex vivo* immune responses to TB-specific antigen (TBAg), have been widely used for detection of Mtb infection worldwide (13–15). However, the most critical limitation is their inability to distinguish active TB from latent tuberculosis infection (LTBI) (16–18). This limitation leads to their low specificity in diagnosing EPTB, especially in low- and middle-income countries (19).

The development of rapid, non-invasive, non-sputum-based biomarker tests for diagnosis of EPTB are still urgently needed, which is also highlighted as a high priority in a recent WHO consensus report (20). Based on analysis of the results of T-SPOT.TB (T-SPOT, one of two commercially available IGRAs) in different situations, we found that T-SPOT results are affected by the immune status of the host. Theoretically, patients with active TB will have high level of TBAg results, while the interpretation of T-SPOT data becomes ambiguous because TBAg results are decreased in immunocompromised patients. However, we found that phytohemagglutinin (PHA) results in T-SPOT assay can reflect the immune status of the patient and are also decreased in this condition. Thus, our group has previously proposed that calculation of the ratio of TBAg to PHA (TBAg/PHA ratio) in T-SPOT assay can eliminate the impact of individual immune heterogeneity on T-SPOT assay, which results in improved performance in distinguishing pulmonary TB from LTBI (21). In this study, we further assessed the use of the TBAg/PHA ratio for diagnosis and treatment monitoring of EPTB.

## Materials and Methods

### Study Subjects

Between October 2012 and March 2017, all clinically suspected EPTB patients who were finally diagnosed as confirmed or probable EPTB and simultaneously did T-SPOT assay were continuously recruited from Tongji Hospital. The clinically suspected EPTB patients who had other diagnoses (e.g., metastatic tumor, bacterial infection, and rheumatoid arthritis) and simultaneously did T-SPOT assay during the same period of time were recruited as non-EPTB controls. The inclusion and diagnostic criteria of EPTB and non-EPTB are shown in Table S1 in Supplementary Material. The suspected EPTB patients with undefined final diagnosis were excluded. To validate the results of this study, we recruited another group of participants who met the same inclusion criteria from Sino-French New City Branch of Tongji Hospital between October 2016 and May 2017. Immunosuppressive conditions were defined as patients with underlying diseases or conditions such as malignancy, chronic renal failure, liver cirrhosis, diabetes mellitus, HIV infection, or patients with solid organ transplantation or rheumatologic disease and receiving immunosuppressive treatment. Patients younger than 18 years of age and those undergoing TB treatment were also excluded. This study was approved by the ethical committee of Tongji hospital, Tongji Medical College, Huazhong University of Science and Technology, Wuhan, China. All participants gave written consent to the study.

### T-SPOT Assay

Peripheral blood T-SPOT assay (Oxford Immunotec, Oxford, England) was performed according to the manufacturer’s instructions. Briefly, the isolated peripheral blood mononuclear cells (PBMCs) (2.5 × 10^5^) were added to 96-well plates precoated with anti-IFN-γ antibody. Four wells were used for each patient: medium well, PHA well, early secreted antigenic target 6 (ESAT-6), and culture filtrate protein 10 (CFP-10) wells. Plates were incubated for 16–20 h at 37 C° with 5% CO_2_ and developed using an anti-IFN-γ antibody conjugate and substrate to detect the presence of secreted IFN-γ. Spot-forming cells (sfc) were counted with an automated ELISPOT reader (CTL Analyzers, Cleveland, OH, USA). To ensure the reliability of T-SPOT assay, the following points need to be considered: (1) PBMCs were isolated within 4 h of blood collection; (2) each new batch of T-SPOT reagent must be validated before use; (3) T-SPOT assay was performed strictly according to the manufacturer’s protocol; and (4) the automated ELISPOT reader was calibrated with reference plate every month.

### Calculation of the TBAg/PHA Ratio

We calculated the ratios of (1) ESAT-6 sfc to PHA sfc and (2) CFP-10 sfc to PHA sfc. The larger of the above two values was defined as the TBAg/PHA ratio of one patient.

### Statistical Analysis

Data were analyzed using GraphPad Prism 5.01 (GraphPad, La Jolla, CA, USA). Differences between groups were analyzed using the Mann–Whitney *U*-test. Receiver operating characteristic (ROC) analysis was performed to determine the best threshold value for distinguishing EPTB from non-EPTB. Area under the curve (AUC) and optimal combination of sensitivity and specificity were reported. Spearman’s rank correlation test for non-parametric data was employed to analyze the relationship between two factors. Statistical significance was determined as *p* < 0.05.

## Results

### Sample Characteristics

Between 2012 and 2017, 734 EPTB patients and 1,137 non-EPTB controls were diagnosed and recruited from Tongji hospital. The demographic and clinical characteristics of the EPTB patients and control individuals are shown in Table 1.

**Table 1:** Demographic and clinical characteristics of EPTB patients and non-EPTB controls.

Characteristic	EPTB patients (*n* = 734)	Non-EPTB controls (*n* = 1,137)
Mean age (mean ± SD), years	43.35 ± 18.05	45.01 ± 14.55
Male sex	439 (59.81)	649 (57.12)
History of tuberculosis	159 (21.66)	43 (3.82)
Disease sites		
Pleural	304 (41.42)	125 (10.99)
Ascitic	59 (8.04)	115 (10.11)
Pericardial	37 (5.04)	112 (9.85)
Central nervous system	85 (11.58)	95 (8.35)
Lymph node	76 (10.35)	128 (11.26)
Bone and joint	57 (7.77)	138 (12.14)
Urinary system	37 (5.04)	175 (15.39)
Genital	34 (4.63)	75 (6.60)
Intestinal	30 (4.09)	116 (10.20)
Skin	15 (2.04)	58 (5.10)
Immunosuppressive conditions^a^	165 (22.48)	284 (24.98)
Confirmed EPTB	301 (41.01)	
Culture	279 (38.01)	
PCR	261 (35.56)	
Probable EPTB	433 (58.99)	
WBC (mean ± SD), ×10^9^/L	6.01 ± 3.21	7.56 ± 3.25
CRP (mean ± SD), mg/L	41.15 ± 38.76	36.59 ± 31.86
ESR (mean ± SD), mm/h	38.25 ± 28.39	32.33 ± 26.91

*Data are presented as numbers (%) unless otherwise indicated*.

*^a^Immunosuppressive conditions were defined as patients with underlying diseases or conditions such as malignancy, chronic renal failure, liver cirrhosis, diabetes mellitus, HIV infection, or patients with solid organ transplantation or rheumatologic disease and receiving immunosuppressive treatment*.

*EPTB, extrapulmonary tuberculosis; PCR, polymerase chain reaction; WBC, white blood cells; CRP, C-reactive protein; ESR, erythrocyte sedimentation rate*.

### The Positive Rate of T-SPOT Assay

The total positive rate of T-SPOT in EPTB patients was 88.15% (Table S2 in Supplementary Material). The positive rate of T-SPOT showed some differences in patients with different forms of EPTB. In general, it was relatively low in patients with serous fluid such as pleural, ascitic, or pericardial TB. The total positive rate of T-SPOT in non-EPTB controls was 32.28%, which was similar to the prevalence of LTBI as reported previously (22).

### Using T-SPOT Results or the TBAg/PHA Ratio for Diagnosis of EPTB

**Figure 1:**
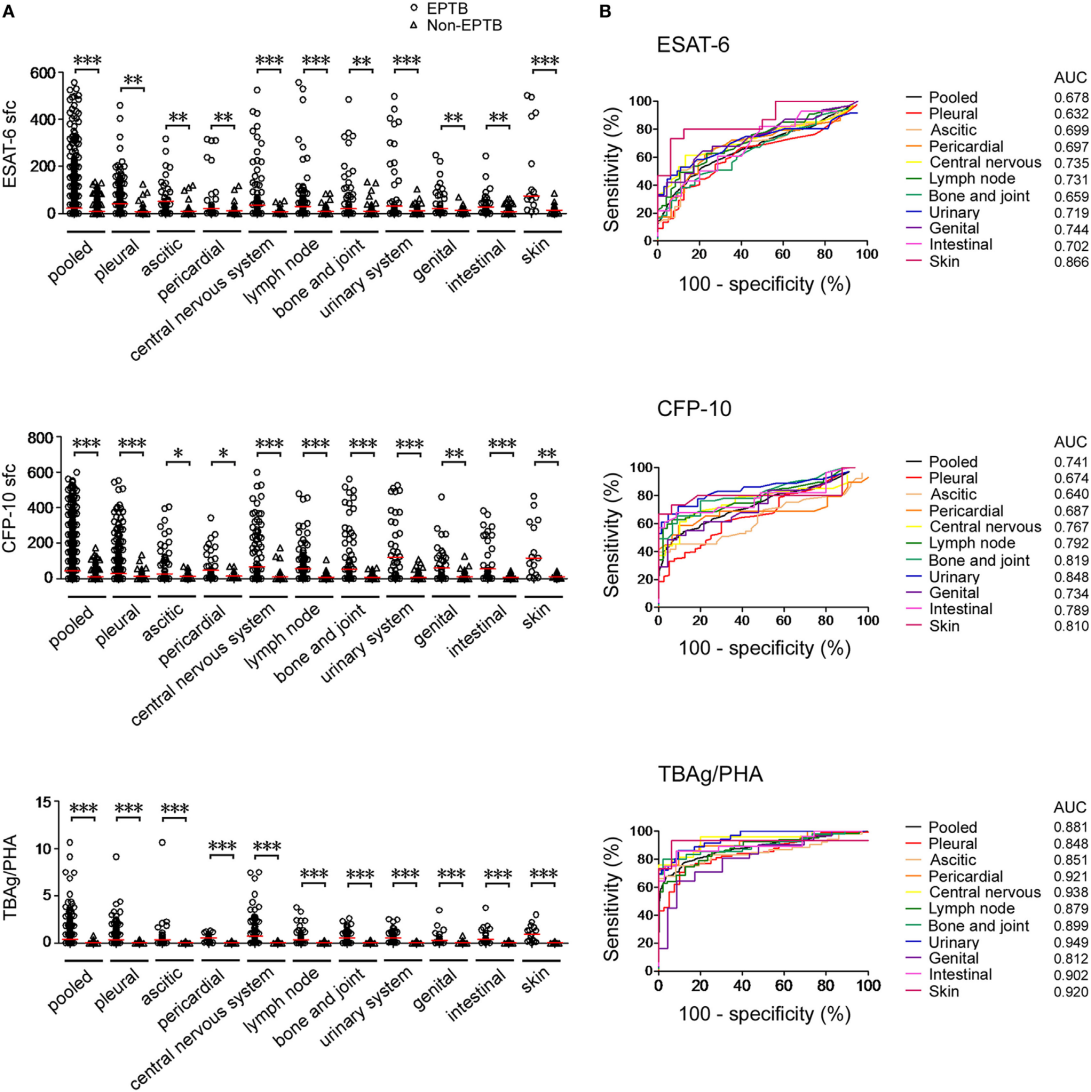

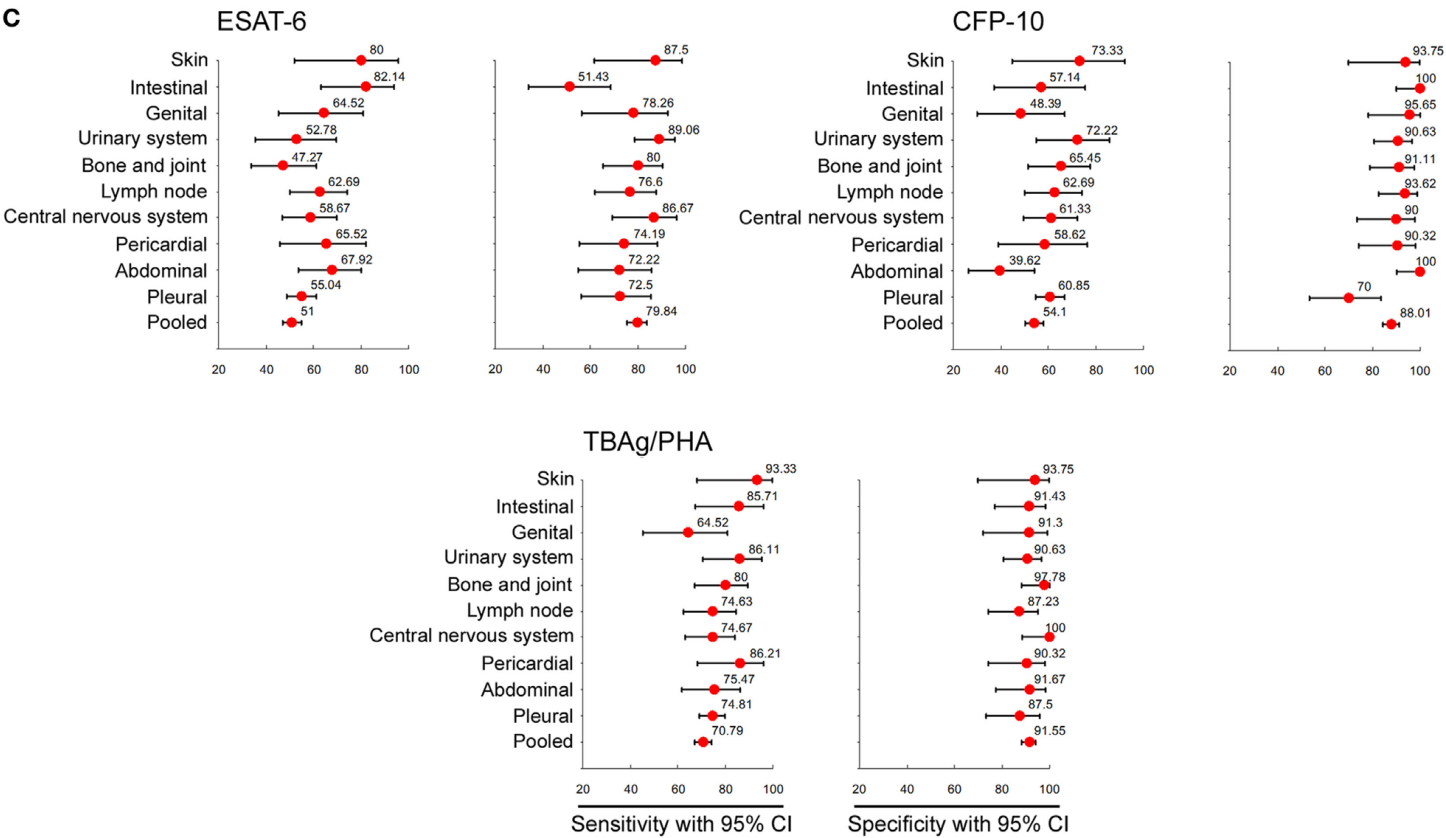
T-SPOT results and the TBAg/PHA ratio in distinguishing EPTB from non-EPTB. **(A)** Dot plots showing ESAT-6 sfc, CFP-10 sfc, and the TBAg/PHA ratio in T-SPOT positive patients with different types of EPTB (*n* = 647) and non-EPTB (*n* = 367). Bars indicate medians (*p < 0.05, **p < 0.01, and ***p < 0.001). **(B)** Receiver operating characteristic analysis was performed for ESAT-6 sfc, CFP-10 sfc, and the TBAg/PHA ratio to determine threshold values for discriminating between EPTB and non-EPTB. **(C)** Forest plots showing the optimal sensitivity and specificity of ESAT-6 sfc, CFP-10 sfc, and the TBAg/PHA ratio in distinguishing EPTB from non-EPTB. Abbreviations: EPTB, extrapulmonary tuberculosis; ESAT-6, early secreted antigenic target 6; CFP-10, culture filtrate protein 10; AUC, area under the curve; CI, confidence intervals; TBAg, TB-specific antigen; PHA, phytohemagglutinin; sfc, spot-forming cells.

In T-SPOT positive patients, although both ESAT-6 and CFP-10 sfc in EPTB patients were significantly higher than those in non-EPTB controls (Figure 1A), ROC analysis showed that the performance of them in distinguishing EPTB from non-EPTB was limited. The pooled AUC for ESAT-6 and CFP-10 sfc were 0.678 and 0.741, respectively (Figure 1B). The sensitivity and specificity were moderate (Figure 1C).

The performance of the TBAg/PHA ratio was evaluated as it can eliminate the impact of individual immune variation on T-SPOT assay as described in our previous study (21). We observed that the TBAg/PHA ratio in all types of EPTB patients was remarkably higher than that in non-EPTB controls (Figure 1A). As expected, a further calculation of the TBAg/PHA ratio yielded improved diagnostic performance compared with directly using ESAT-6 or CFP-10 sfc (Figure 1B). The pooled AUC for the TBAg/PHA ratio in distinguishing EPTB from non-EPTB was 0.881. If using the threshold value of 0.20, the sensitivity and specificity were 70.79 and 91.55%, respectively (Figure 1C).

### The TBAg/PHA Ratio in Confirmed and Probable EPTB Patients

**Figure 2:**
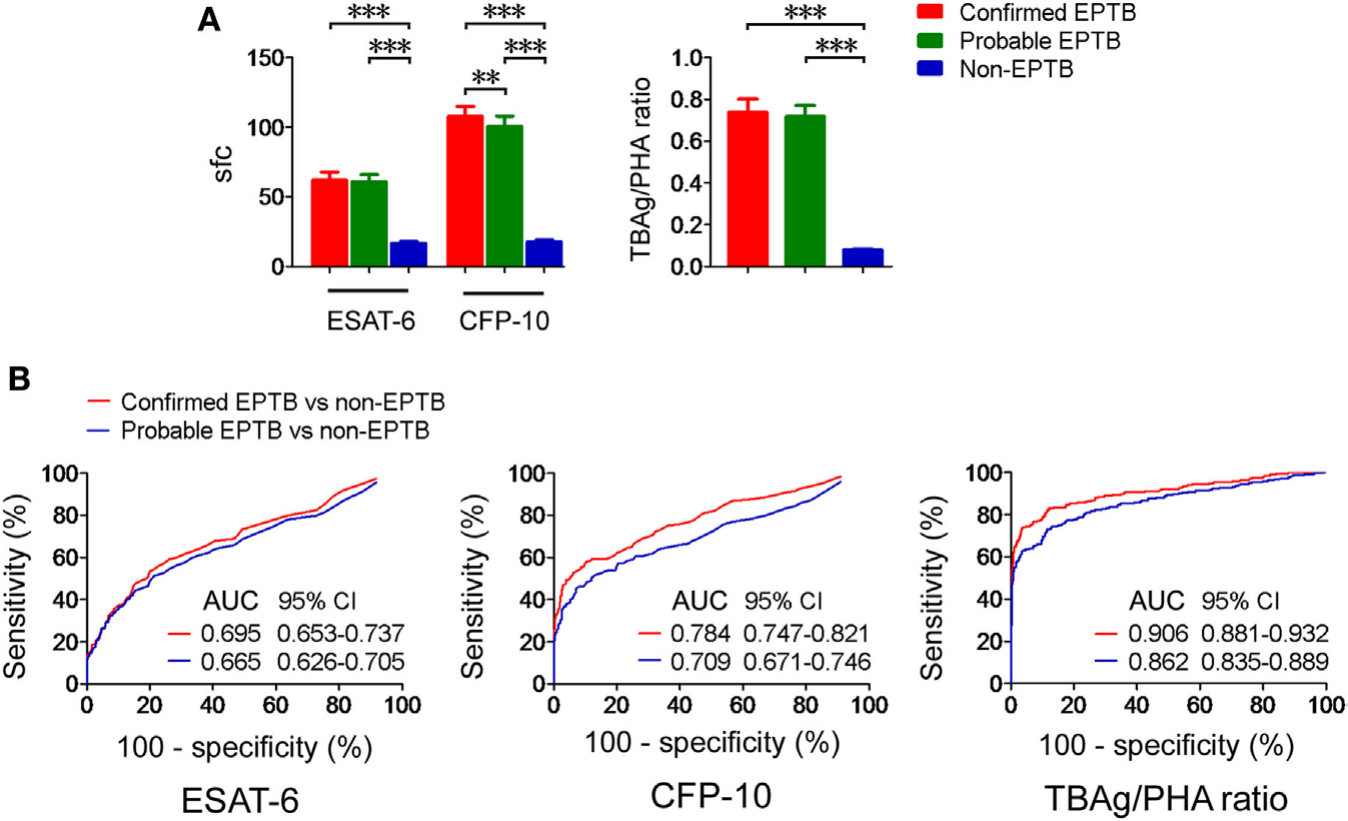
The TBAg/PHA ratio in confirmed and probable EPTB patients. **(A)** Histograms showing ESAT-6 sfc, CFP-10 sfc, and the TBAg/PHA ratio in T-SPOT positive patients with confirmed EPTB (*n* = 273), probable EPTB (*n* = 374), and non-EPTB (*n* = 367). Data are shown as the mean ± SEM (**p < 0.01 and ***p < 0.001). **(B)** Receiver operating characteristic analysis was performed for ESAT-6 sfc, CFP-10 sfc, and the TBAg/PHA ratio in distinguishing between confirmed EPTB and non-EPTB or between probable EPTB and non-EPTB. Abbreviations: EPTB, extrapulmonary tuberculosis; ESAT-6, early secreted antigenic target 6; CFP-10, culture filtrate protein 10; AUC, area under the curve; CI, confidence intervals; TBAg, TB-specific antigen; PHA, phytohemagglutinin; sfc, spot-forming cells.

We next analyzed the TBAg/PHA ratio in confirmed and probable EPTB patients with positive T-SPOT results. Our results showed that except for CFP-10 sfc, both ESAT-6 sfc and TBAg/PHA ratio had no significant difference between confirmed and probable EPTB patients. However, all these results in either confirmed or probable EPTB patients were significantly higher than those in non-EPTB controls (Figure 2A). Furthermore, ROC analysis showed that the AUCs of ESAT-6, CFP-10, and TBAg/PHA ratio in distinguishing confirmed EPTB from non-EPTB were slightly higher than those in distinguishing probable EPTB from non-EPTB (Figure 2B). These data suggest that the diagnostic accuracy of the TBAg/PHA ratio in confirmed EPTB patients is slightly better than that in probable EPTB patients.

### Validation of the TBAg/PHA Ratio

**Figure 3:**
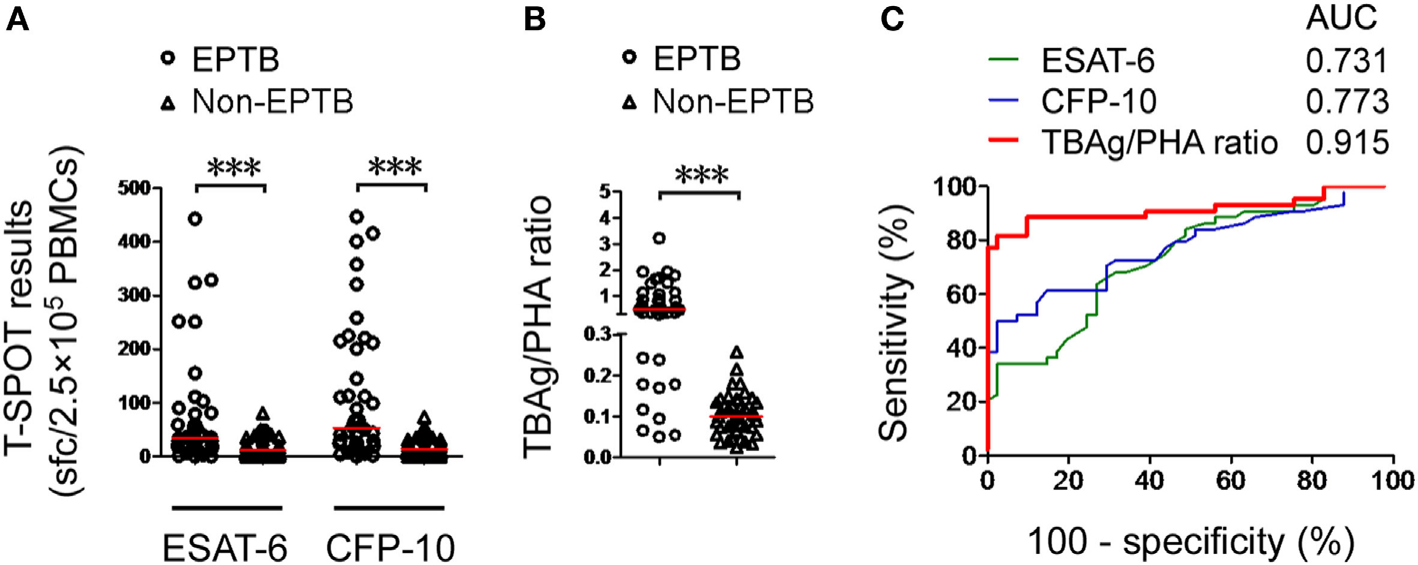
Validation of the TBAg/PHA ratio. **(A)** Dot plots showing ESAT-6 sfc, CFP-10 sfc, and **(B)** the TBAg/PHA ratio in T-SPOT positive validation patients with EPTB (*n* = 44) and non-EPTB (*n* = 41). Bars indicate medians (***p < 0.001). **(C)** Receiver operating characteristic analysis was performed for ESAT-6 sfc, CFP-10 sfc, and the TBAg/PHA ratio in distinguishing EPTB from non-EPTB. Abbreviations: EPTB, extrapulmonary tuberculosis; ESAT-6, early secreted antigenic target 6; CFP-10, culture filtrate protein 10; AUC, area under the curve; TBAg, TB-specific antigen; PHA, phytohemagglutinin; sfc, spot-forming cells.

To validate the results of this study, we recruited another 44 EPTB patients and 41 non-EPTB controls who met the same inclusion criteria and had positive T-SPOT results from Sino-French New City Branch of Tongji Hospital. Given that the performance of the TBAg/PHA ratio was relatively low in pleural and ascitic TB, these two types of EPTB patients were excluded in the validation population. Similarly, the performance of directly using T-SPOT results in distinguishing EPTB from non-EPTB was limited (Figures 3A,C). The TBAg/PHA ratio showed an obvious improvement in distinguishing these two conditions (Figures 3B,C). The AUC of the TBAg/PHA ratio was 0.915, which is higher than the AUC value obtained in our original study. Using 0.24 as the threshold value of the TBAg/PHA ratio, the sensitivity and specificity were 81.82 and 97.56%, respectively. These data suggest that the TBAg/PHA ratio is useful in the diagnosis of most types of EPTB, except for pleural and ascitic TB.

### The TBAg/PHA Ratio in EPTB Patients With Different Immune Status

**Figure 4:**
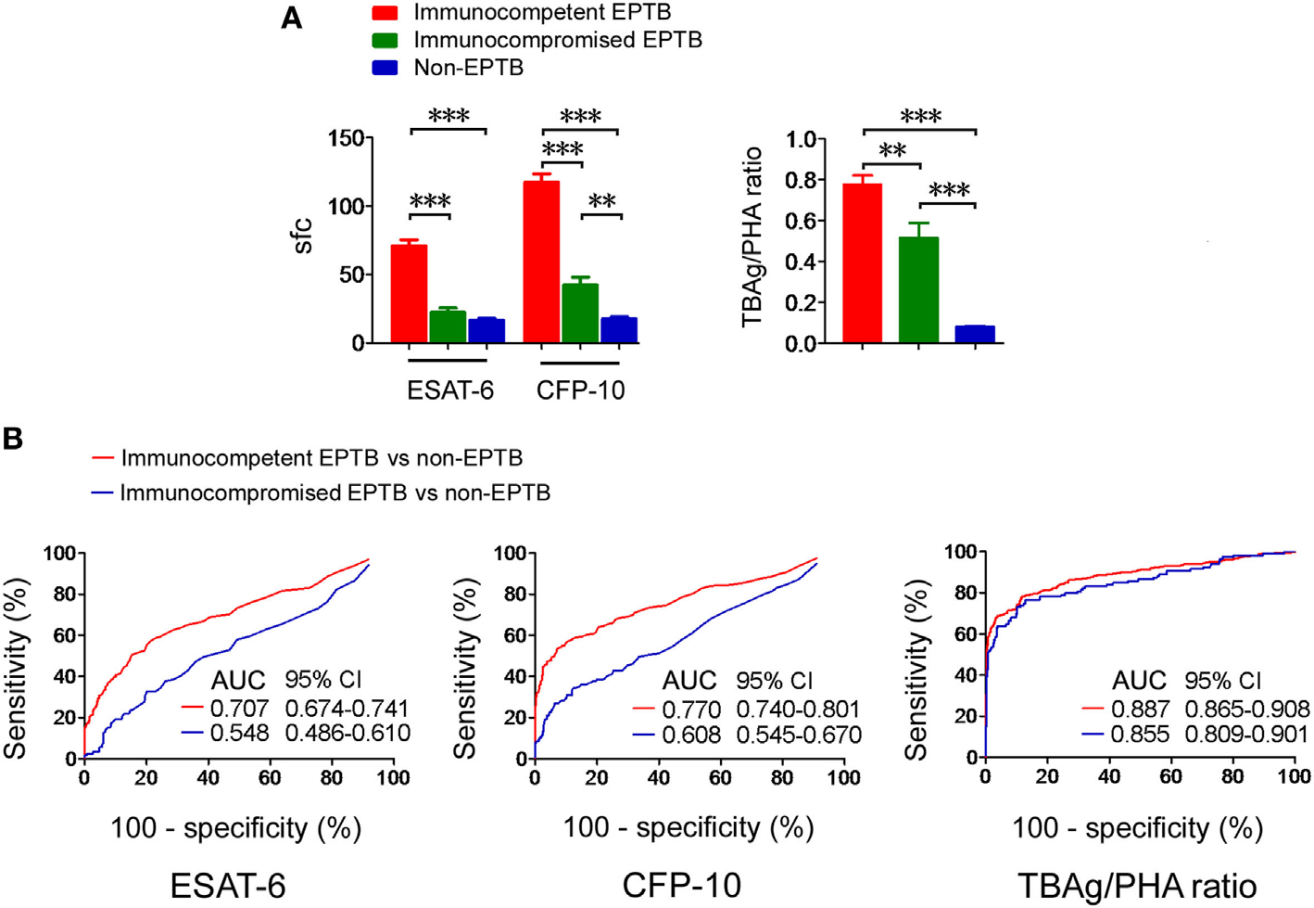
T-SPOT results and the TBAg/PHA ratio in patients with different immune status. **(A)** Histograms showing ESAT-6 sfc, CFP-10 sfc, and the TBAg/PHA ratio in immunocompetent EPTB patients (*n* = 528), immunocompromised EPTB patients (*n* = 119), and non-EPTB controls (*n* = 367) with positive T-SPOT results. Data are shown as the mean ± SEM (**p < 0.01 and ***p < 0.001). **(B)** Receiver operating characteristic analysis was performed for ESAT-6 sfc, CFP-10 sfc, and the TBAg/PHA ratio in distinguishing between immunocompetent EPTB and non-EPTB or between immunocompromised EPTB and non-EPTB. Abbreviations: EPTB, extrapulmonary tuberculosis; ESAT-6, early secreted antigenic target 6; CFP-10, culture filtrate protein 10; AUC, area under the curve; CI, confidence intervals; TBAg, TB-specific antigen; PHA, phytohemagglutinin; sfc, spot-forming cells.

The T-SPOT results in EPTB patients with different immune status were assessed. We observed that both T-SPOT results and TBAg/PHA ratio were significantly decreased in immunocompromised patients compared with immunocompetent patients (Figure 4A). ROC analysis showed that the performance of ESAT-6 and CFP-10 sfc in distinguishing immunocompetent EPTB from non-EPTB was better than that in distinguishing immunocompromised EPTB from non-EPTB (Figure 4B). However, this trend was not evident for the performance of the TBAg/PHA ratio. These data indicate that the TBAg/PHA ratio is less affected by the immune status of the host.

### PHA sfc Reflects the Immune Status and Correlates With the Results of T-SPOT

**Figure 5:**
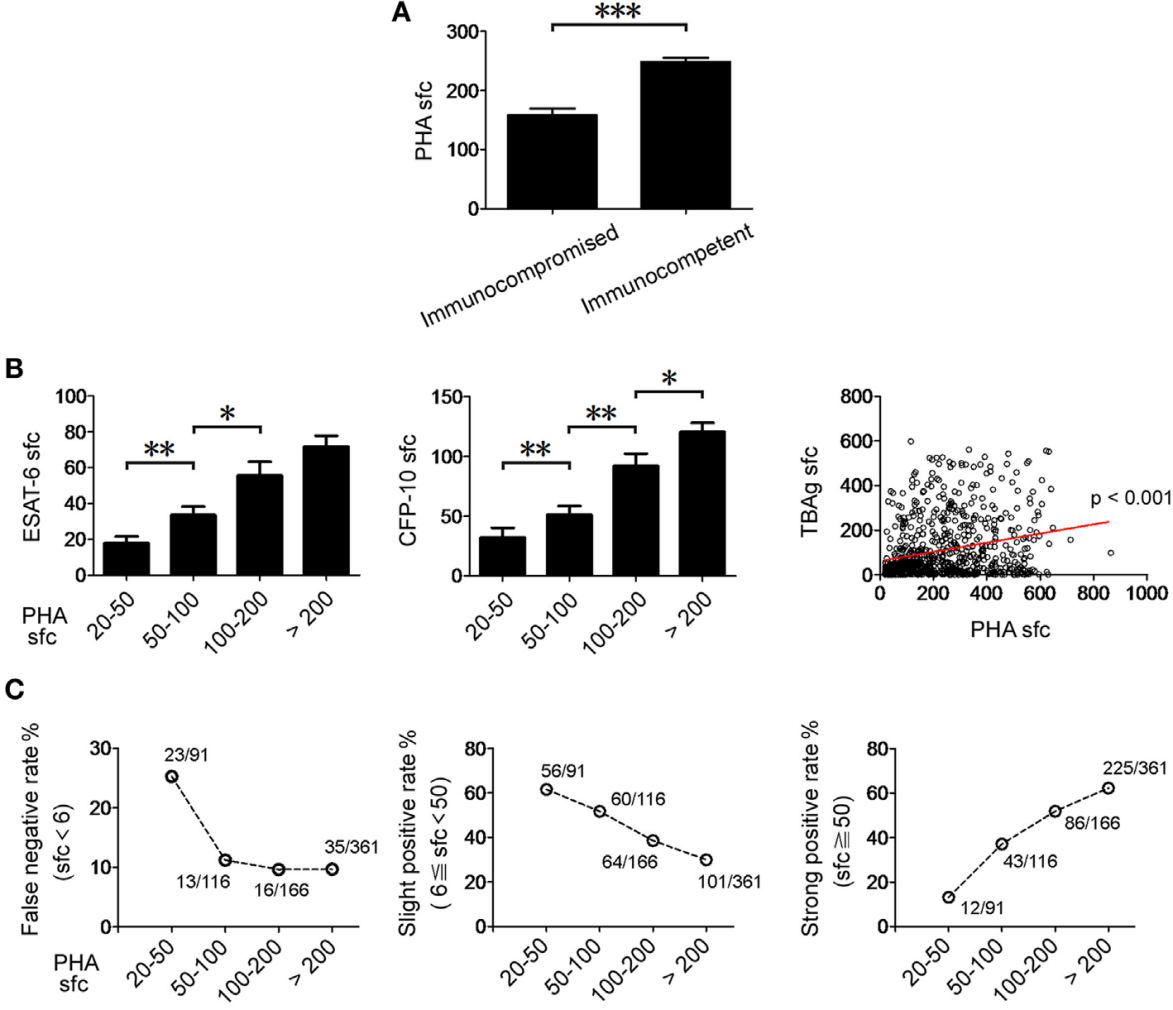
The relationship between PHA sfc and T-SPOT results in EPTB patients. **(A)** Histograms showing PHA sfc in immunocompetent (*n* = 528) and immunocompromised (*n* = 119) EPTB patients. **(B)** Histograms showing ESAT-6 and CFP-10 sfc in EPTB patients with different PHA categories (PHA sfc 20–50, *n* = 91; PHA sfc 50–100, *n* = 116; PHA sfc 100–200, *n* = 166; PHA sfc > 200, *n* = 361). Data are shown as the mean ± SEM (*p < 0.05, **p < 0.01, and ***p < 0.001). Correlation between PHA sfc and TBAg sfc (the large of ESAT-6 and CFP-10 sfc) (Spearman’s rank correlation test). **(C)** The percentages of false negative (TBAg sfc < 6, *n* = 87), slight positive (6 ≦ TBAg sfc < 50, *n* = 281), and strong positive (TBAg sfc ≧ 50, *n* = 366) T-SPOT results in EPTB patients with different PHA categories are shown. Abbreviations: EPTB, extrapulmonary tuberculosis; ESAT-6, early secreted antigenic target 6; CFP-10, culture filtrate protein 10; PHA, phytohemagglutinin; TBAg, TB-specific antigen; sfc, spot-forming cells.

By comparing the clinical characteristics of EPTB patients with false negative (sfc < 6), slight positive (6 ≦ sfc < 50), or strong positive (sfc ≧ 50) T-SPOT results, we confirmed that the immunosuppressive conditions are indeed the major factors that contribute to decreased or even false negative T-SPOT results (Table S3 in Supplementary Material). Actually, interpretation of T-SPOT results in immunocompromised patients becomes ambiguous, as it is very difficult to distinguish TB disease from latent infection in this condition. Interestingly, the PHA sfc in immunocompromised EPTB patients was significantly lower than that in immunocompetent EPTB patients (Figure 5A). Furthermore, there was a significant correlation between PHA sfc and TBAg sfc (Figure 5B).

We unexpectedly found that the false negative rate of T-SPOT was over 25% in low PHA sfc group (PHA sfc: 20–50). The slight positive rate of T-SPOT was gradually decreased with the increase of PHA sfc, and over half of the EPTB patients had slight positive T-SPOT results which are usually attributed to LTBI in relatively low PHA sfc group (PHA sfc: 20–100) (Figure 5C). These data suggest that PHA sfc can reflect the immune status of the host and help to judge the credibility of T-SPOT results.

### The TBAg/PHA Ratio Responds to Anti-TB Treatment

**Figure 6:**
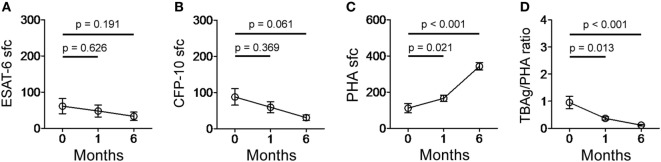
T-SPOT results and the TBAg/PHA ratio in EPTB patients after anti-TB treatment. Dot plots showing **(A)** ESAT-6 sfc, **(B)** CFP-10 sfc, **(C)** PHA sfc, and **(D)** the TBAg/PHA ratio in EPTB patients (*n* = 13) before and after 1 and 6 months of anti-TB treatment. Abbreviations: EPTB, extrapulmonary tuberculosis; ESAT-6, early secreted antigenic target 6; CFP-10, culture filtrate protein 10; PHA, phytohemagglutinin; TBAg, TB-specific antigen; sfc, spot-forming cells.

The quantitative change of the TBAg/PHA ratio during anti-TB treatment might reflect therapeutic efficacy. We performed serial T-SPOT assay on 13 EPTB patients before and after 1 and 6 months of anti-TB treatment. Although both ESAT-6 and CFP-10 sfc were gradually decreased after anti-TB treatment, statistical analysis showed there was no significant difference between before and after treatment (Figures 6A,B). By contrast, PHA sfc was significantly increased even after 1 month of anti-TB therapy. Thus, the calculated TBAg/PHA ratio was more significantly decreased after 1 month of anti-TB treatment, and this ratio was continuously reduced to a very low level after 6 months of treatment (Figures 6C,D).

## Discussion

Extrapulmonary tuberculosis contributes significantly to TB-related morbidity and can cause serious consequences for patients (5). But unfortunately, the diagnosis of EPTB is often delayed or even missed because of insidious clinical presentation and poor performance of diagnostic tests (23). In this study, we introduce a rapid, non-invasive, and non-sputum-based method which based on calculation of the TBAg/PHA ratio of T-SPOT assay for diagnosis and treatment monitoring of EPTB.

The sensitivity of T-SPOT assay seems to be satisfied in most types of EPTB (24–27). The dilemma of this method is limited specificity in high TB-burden areas due to its inability to discriminate TB disease from LTBI (24, 28). As a result, the specificity of T-SPOT in the diagnosis of EPTB is dependent upon the local prevalence of LTBI. As described in our previous study (21), the following two major factors may contribute to the difficulty of directly using T-SPOT results in distinguishing active TB from LTBI. First, LTBI individuals with robust immune systems may have a relatively high TBAg sfc. Second, active TB patients with immunocompromised status will have a relatively low TBAg sfc. However, PHA sfc in T-SPOT assay can reflect the immune status of the host and will correspondingly increase or decrease as well. Thus, calculating the TBAg/PHA ratio can eliminate the impact of individual immune variation on T-SPOT assay, which is the most important reason why calculation of the TBAg/PHA ratio is better than directly using T-SPOT results in the diagnosis of EPTB.

Whether the host’s immune status can affect the T-SPOT results in EPTB patients is controversial (24, 26, 29). In general, the sensitivity of the T-SPOT is decreased in immunocompromised EPTB patients. The debate is whether this decreased trend has statistical significance. We have compared the T-SPOT results in patients with different immune status and found that the performance of T-SPOT in distinguishing EPTB from non-EPTB was indeed affected by the immune status of the host. However, the TBAg/PHA ratio was less affected by the immune status, which might contribute to the improved performance of it in the diagnosis of EPTB.

The understanding of T-SPOT results in patients with immunosuppression is sometimes difficult in clinical practice. We confirmed that the immunosuppression status was the important factor to cause slight positive or even negative T-SPOT results in EPTB patients. Interestingly, PHA results in T-SPOT assay reflected the immune status and might help to solve the problem to a certain extent. We unexpectedly found that a high false negative rate of T-SPOT was existed in low PHA category, which suggests that EPTB should be still suspected in patients who had negative T-SPOT results but with low PHA sfc. Furthermore, slight positive T-SPOT results which are usually attributed to LTBI (16) should be highly suspected of having EPTB because most of the EPTB patients had slight positive T-SPOT results in low PHA group. Importantly, PHA sfc reflects immune status and provides additional information for a better understanding of the T-SPOT results.

Given that PHA sfc in T-SPOT is used to calculate the TBAg/PHA ratio and also can be used to assess the immune status, how to get accurate PHA results is very important. Except for the precautions described above such as reagent validation, standard operating procedures, and ELISPOT reader calibration, the following two points should be especially noted: (1) the cell count should be accurate and no more than 2.5 × 10^5^ PBMCs are added to one well. If an excessive number of PBMCs are added to T-SPOT well, it is difficult to count PHA sfc accurately because too many spots are crowded in one well; and (2) the substrate incubation time should be strictly limited to 7 min. If it is prolonged, the single PHA spot will link together and it is impossible to count the PHA sfc accurately. If all steps are performed correctly, the reproducibility of the PHA results is acceptable. We have repeatedly performed T-SPOT by using peripheral blood samples from the same patients, and the coefficient of variation of PHA sfc was below 15%.

There are several advantages for using the TBAg/PHA ratio to diagnose EPTB. First, it is a rapid and non-invasive method for diagnosis of EPTB just on the basis of T-SPOT assay, without needing for combination with other methods or special equipments. Second, the TBAg/PHA ratio shows high specificity in the diagnosis of EPTB, which is of great importance in TB-endemic countries because direct T-SPOT results can’t distinguish TB disease from LTBI in these areas. Third, T-SPOT assay has been widely used in many laboratories worldwide. Thus, we need not to introduce a new method and just add a calculation of the existed results. Several limitations of this study should be mentioned. First, this is a case-control retrospective study. Second, the number of patients with pleural TB is significantly higher than the number of those with other types of EPTB. However, this is in accordance with previous report showing that the most common form of EPTB is pleural TB (4). Furthermore, the performance of the TBAg/PHA ratio is decreased in pleural and ascitic TB compared with other types of EPTB, which results in unsatisfactory diagnostic efficacy (AUC < 0.9) of this method in pooled EPTB. Third, less than half of the EPTB patients are confirmed by microbiological evidence, which might be caused by the pauci-bacillary nature of disease. Fourth, the TBAg/PHA ratio could be used in only T-SPOT positive EPTB patients. Although the positive rate of T-SPOT in EPTB patients is high, there is still a small percentage of EPTB patients with negative T-SPOT results.

Taken together, we introduce a new use of T-SPOT assay by calculation of the TBAg/PHA ratio. This method might have the potential to be used for diagnosis and treatment monitoring of EPTB.

## Ethics Statement

This study was approved by the ethical committee of Tongji hospital, Tongji Medical College, Huazhong University of Science and Technology.

## Author Contributions

FW, JY, YZ, YL, and SW performed experiments and analyzed data; FW, MH, BY, JH, LM, and ZS developed the concept, designed the study, analyzed data, and wrote the paper; all the authors commented on the paper.

## Conflict of Interest Statement

The authors declare that the research was conducted in the absence of any commercial or financial relationships that could be construed as a potential conflict of interest.
